# Benchmarking experience to improve paediatric healthcare: listening to the voices of families from two European Children’s University Hospitals

**DOI:** 10.1186/s12913-021-06094-z

**Published:** 2021-01-27

**Authors:** Ilaria Corazza, Kendall Jamieson Gilmore, Francesca Menegazzo, Valts Abols

**Affiliations:** 1 Management and Healthcare Laboratory (MeS Lab), Institute of Management and EMbeDS, Sant’Anna School of Advanced Studies, Piazza Martiri della Libertà 33, 56127 Pisa, Italy; 2 Azienda Ospedaliero Universitaria Meyer, Viale Pieraccini 24, 50139 Florence, Italy; 3 Children’s Clinical University Hospital, Vienības gatve 45, Rīga, LV-1004 Latvia

**Keywords:** Benchmarking, Patient reported experience measures, Paediatric care

## Abstract

**Background:**

Patient Reported Experience Measures (PREMs) are recognized as an important indicator of high quality care and person-centeredness. PREMs are increasingly adopted for paediatric care, but there is little published evidence on how to administer, collect, and report paediatric PREMs at scale.

**Methods:**

This paper describes the development of a PREMs questionnaire and administration system for the Meyer Children’s University Hospital in Florence (Meyer) and the Children’s Clinical University Hospital in Riga (CCUH). The system continuously recruits participants into the electronic administration model, with surveys completed by caregivers or adolescents at their convenience, post-discharge. We analyse 1661 responses from Meyer and 6585 from CCUH, collected from 1st December 2018 to 21st January 2020. Quantitative and qualitative experience analyses are included, using Pearson chi-square tests, Fisher’s exact tests and narrative evidence from free text responses.

**Results:**

The large populations reached in both countries suggest the continuous, digital collection of paediatric PREMs described is feasible for collecting paediatric PREMs at scale. Overall response rates were 59% in Meyer and 45% in CCUH. There was very low variation in mean scores between the hospitals, with greater clustering of Likert scores around the mean in CCUH and a wider spread in Meyer for a number of items. The significant majority of responses represent the carers’ point of view or the perspective of children and adolescents expressed through proxy reporting by carers.

**Conclusions:**

Very similar reported scores may reflect broadly shared preferences among children, adolescents and carers in the two countries, and the ability of both hospitals in this study to meet their expectations.

The model has several interesting features: inclusion of a narrative element; electronic administration and completion after discharge from hospital, with high completion rates and easy data management; access for staff and researchers through an online platform, with real time analysis and visualization; dual implementation in two sites in different settings, with comparison and shared learning. These bring new opportunities for the utilization of PREMs for more person-centered and better quality care, although further research is needed in order to access direct reporting by children and adolescents.

**Supplementary Information:**

The online version contains supplementary material available at 10.1186/s12913-021-06094-z.

## Background

Patient experience is recognized as an important measure of performance in healthcare; not just as a ‘nice to have’ in addition to good clinical care, but an integral element of delivering quality healthcare [1]. As well as a worthy aim in itself, there is evidence that better patient experiences are associated with a number of desirable effects: positive patient behaviors, including adherence to clinical advice and treatments; better clinical outcomes; and reduced unnecessary healthcare usage [2]. These findings have been recorded elsewhere, alongside consistent positive associations between patient experience, safety, and clinical effectiveness [3].

Providing a positive patient experience is increasingly a core aim at many levels in health systems, and is one of the features in the Quadruple Aim, a model consisting of achieving sustainable cost, better population health, improved patient experience and improved provider experience [4]. Additionally, patient experience data can be used alongside other data to better measure and facilitate value-based care at population levels [5–7]. Patient experience is widely measured using Patient Reported Experience Measures (PREMs), which capture different elements of what happened in hospital (e.g. communication at discharge, ward environment) and can be used to recreate what a patient experienced during their interactions with healthcare providers as well as their overall perception of care [8].

This increased focus on PREMs is not evenly spread, with some populations or clinical areas not typically included in PREMs collection [9]. One such area is paediatric care, which, despite internationally agreed statements on the rights of children to be consulted and involved in decision making, and agreement by health services researchers of the need to include children’s views, remain an underrepresented group [10, 11]. There are several possible reasons for this: a residual view that children are not sufficiently developed to be able to meaningfully contribute [12]; comparatively little evidence on the best mechanisms for collecting paediatric experience data [10]; and, perceived and real challenges in enabling children to adequately describe their experiences to adults collecting such data. However, studies have shown that even young children have the capacity to understand their condition and care [13], and children’s comments are coherent and pertinent to hospitalization experiences [11, 14]. Additionally, there are comparatively few paediatric hospitals (where dedicated paediatric PREMs are more likely to be feasible) compared to generalist hospitals, which may further limit the availability of paediatric PREM data.

Taken together, this dearth of paediatric patient experience data could have a number of potentially detrimental effects: a reduced understanding of what children and adolescents in hospital (and their caregivers) value; missed opportunities for providing more patient-centred care; and a lack of understanding about the relationship between experience, outcomes, and ways of working in healthcare settings.

In particular, an inability to record, benchmark and compare performance on shared paediatric experience measures removes a set of levers to improve provider performance. Benchmarking and disclosure is known to be an effective means to improve health care [15], and the opportunities to do so for patient reported measures were highlighted recently by the Organisation for Economic Co-operation and Development (OECD), which emphasized the need to conduct international benchmarking of PREMs [9]. To benchmark effectively requires sufficient data, which is comparable, relevant, timely and actionable. For paediatric care, this is a particular challenge for the reasons stated above, leading to lost opportunities to learn from different providers and systems, and to improve the quality of care for children and adolescents in hospital.

This paper describes a model for and results from collecting and comparing paediatric PREMs in Italy and Latvia. In doing so, it explores the feasibility of establishing an international PREMs benchmarking observatory, making use of digital and continuous collection using a shared system and support function. The results from the two areas are compared and evaluated, to provide a picture of the ways in which children and adolescents in hospital and their caregivers do or do not provide the same responses at international level.

The aims of this work are to:

1. explore the feasibility of establishing an international benchmarking system for PREMs, starting with specialist paediatric hospitals in two countries, Italy and Latvia; and
2. compare responses between the two hospitals and conduct international benchmarking of paediatric PREMs.

## Methods

### Setting

The study was conducted in the Meyer Children’s University Hospital in Florence (Meyer), Italy, and the Children’s Clinical University Hospital in Riga (CCUH), Latvia. Both are specialist paediatric hospitals offering the full range of paediatric emergency and inpatient services.

In Florence, the paediatric PREMs programme was developed as an extension to the ongoing collection of adult PREMs in several hospitals in the surrounding Tuscany region and more widely. In Riga, the pilot collection of paediatric PREMs was implemented as part of an European Commission Structural Reform Support Service (SRSS) to develop a Health System Performance Assessment (HSPA) system in Latvia [16]. The Children’s Clinical University Hospital elected to undertake the first PREMs collection and reporting in Latvia through that reform initiative. Both programmes are delivered with support from the Management and Healthcare Laboratory (MeS Lab) of the Sant’Anna School of Advanced Studies in Pisa.

### Institutional context

Meyer and CCUH use different diagnosis-related group (DRG) systems, preventing direct case-mix comparison. More generally, both hospitals attract patients from large territories and treat complex cases.

Meyer is a first level referral center for the local catchment area, and also attracts regional, extra-regional and international patients through its role as referral center in the Regional Paediatric Network, and through its specializations recognized nationally and internationally. Overall, 26% of Meyer patients in the past five years have been from outside the Tuscany region.

CCUH is the only tertiary hospital for children in Latvia providing multidisciplinary care in more than 40 specialties, and educational and science programs for health professionals. CCUH serves as ultimate referral center for children from all Latvia. CCUH is an expert in developing and improving health literacy for children’s health care in Latvia.

Further information about the respective institutional contexts, is provided in Table 1:

**Table 1:** Institutional context of Meyer and CCUH

	Meyer	CCUH
Ownership and structure	• Publicly-held • 250 multi-specialist beds • Around 1400 staff	• Publicly-held • 300 multi-specialist beds • Around 1920 staff
Services	• Emergency medical services and observation • Stationary medical care • Ambulatory medical care • Daytime stationary • Diagnostic services and prophylaxis • Rehabilitation services • Rare diseases reference and coordination centre of the Tuscany Region	• Emergency medical services and observation • Stationary medical care • Ambulatory medical care • Daytime stationary • Diagnostic services and prophylaxis • Rehabilitation services • Rare disease coordination centre • Sports medical centre • Epilepsy and sleep medicine centre • Children’s vaccination centre
Research	• Collaborating with the University of Florence	• Collaborating with the Riga Stradins University and the Medical Faculty of Latvian State University
Networks	• (Regional) Paediatric Network of Tuscany Region • (National) Italian Association of Paediatric Hospitals • (International) European Children’s Hospital Organisation, European Reference Networks for rare diseases, a number of partnerships with other healthcare and academic institutions, e. g. Children’s Hospital Association, Children’s Hospital of Philadelphia and Boston Children’s Hospital	• (International) European Children’s Hospital Organisation, Planetree International, European Reference Networks for rare diseases

### Survey administration and data management

The survey is conducted digitally. During the hospital stay, all patients and carers are informed about the PREMs programme by ward staff, and carers are invited to provide contact details upon discharge from the ward on patients’ behalf. There are no exclusion criteria for participants (both patients and carers) in the survey, as one of the main purposes of this study is to test the feasibility of implementing a census-like survey of paediatric PREMs at the hospital level. More specifically, Meyer takes an opt-in enrolment approach while CCUH takes an opt-out enrolment approach. Patients who consent have a flag included in their electronic record, which are then accessed by the MeS Lab servers through an Application Programming Interface (API). This provides a continuous data flow between the PREMs administration system and participating hospitals. This data flow includes: admitting and discharging wards; date of admission and discharge; citizenship, sex and age of the paediatric patient. The process is conducted automatically and anonymously. Contact data are deleted or encrypted after recall or questionnaire completion, whichever is the first.

Within 24 h of discharge, enrolled carers are automatically sent a text message or email containing a unique link through which to access the survey. A reminder is sent 24 h later if the survey has not been accessed.

Survey responses are securely collated, analysed and visualised in real time through a web platform. Each hospital is able to review their quantitative data in aggregate form, with qualitative comments included in a separate feed. Through the web platform, the data can be segmented and viewed in various ways, for example by time period or by discharging ward. Professionals can thereby consult the data related to their own patients, not only at hospital level.

### Questionnaire characteristics

To develop a PREMs survey for use in paediatric settings, the standard Tuscan adult survey - based on a longstanding PREMs survey used in the region, initially based on items from the Picker Institute [6] - was adapted by researchers at the MeS Lab, including through addition of items from the Consumer Assessment of Healthcare Providers and Systems (CAHPS) Child Hospital Survey which has been previously validated, including in international settings [17]. The surveys were then revised and further developed with hospital teams to reflect the local context. Most items are very similar to - or are translated items from - previously validated surveys. The resultant surveys are of differing lengths (63 questions in Riga, 55 in Florence), contain a majority of identical items, and contain the same sections capturing the patient journey:

A. Hospital admission;
B. Child’s/Adolescent’s hospitalization experience;
C. Caregiver’s experience;
D. Hospital environment;
E. Discharge;
F. Overall evaluation;
G. Post-hospitalization phase; and
H. Caregiver’s characteristics.

The primary difference between the two surveys is that the questionnaire addressed to CCUH patients and caregivers includes questions for measuring their experience in the Emergency Department, while the questionnaire addressed to Meyer patients and caregivers does not. Comparison of scores is therefore possible for most, but not all, items.

The questionnaires seek to highlight the voice of children and adolescents, including a section for completion directly by patients, and the opportunity for those older than 13 years to complete the majority of the survey autonomously.

Both surveys contain several open-ended questions, addressed to both children and adolescents directly and to caregivers, to enable them comment in more detail and more widely than typical closed-ended experiential questions allow.

For a full view of questions and response options/scales, please see the questionnaire reported online as additional material.

### Analysis of responses

#### Quantitative analysis

Statistical quantitative analysis was performed in Stata 15, using experience data collected from 1st December 2018 to 21st January 2020.

Confirmatory factor analysis was conducted on relevant survey sections for the two hospitals’ PREM survey responses separately and together, with items mapping onto factors as expected and with small variation in loadings between CCUH and Meyer.

Pearson chi-squared analysis was used to compare participants in Florence and Riga with respect to the type of respondent to the questionnaire and their sociodemographic characteristics; age, citizenship, level of education and employment status.

Fisher’s exact analysis was used to compare distribution of scores for each comparable question between Meyer and Riga hospitals, including *p*-values.

Five survey domains were included in the analysis: experience of children and adolescents, caregiver’s experience, ward admission and comfort, discharge phase and overall evaluation. The dimensions and number of question items populating each domain are reported in Table 2.

**Table 2:** Domains and dimensions of the questionnaires for Meyer and CCUH patients and caregivers

Domain	Dimension	Number of question items
Experience of children and adolescents	‘patient-provider communication’ ‘respect and dignity’ ‘fears and anxiety’ ‘pain’ ‘involvement’	13
Caregiver’s experience	‘caregiver-provider communication’ ‘fears and anxiety’ ‘privacy’ ‘involvement’ ‘information’ ‘team work’	8
Ward admission and comfort	‘kindness and courtesy at the admission’ ‘cleanliness’ ‘silence’ ‘food’ ‘entertainment’	4
Discharge phase	‘information’ ‘training’	9
Overall evaluation	‘care’ ‘child’s health’	2

Since the proportion of adolescents in the responder’s population who completed the questionnaire autonomously is lower than 3% in both hospitals and for other respondents it is not possible to state the degree of autonomy of the child in the response process, the analysis was conducted on all data together, so that the responses provided by children and adolescents themselves and their caregivers are reported jointly.

### Qualitative analysis: anecdotal evidence

There are six open-ended questions in the survey used at CCUH and five open-ended questions in the survey used at Meyer. These sections are presented to respondents as space for ‘storytelling’.

Open-ended questions provide richer and more subjective information than closed-ended questions about selected dimensions of the patient journey: ward admission, hospitalization experience of children and adolescents, caregiver’s point of view, ward environment and staff evaluation.

In the survey used at CCUH the additional open-ended question addresses child, adolescent and caregiver experience during their stay in the emergency department.

An interpretative grid was created by extracting three scaled judgments (negative, intermediate, positive) for each of the five areas of storytelling, eliminating the extreme judgments (of which there were few).

## Results

### Participants’ characteristics

In Florence, 2834 caregivers who gave consent to participate in the survey were contacted either by e-mail or text message, with 14,568 contacted during the same period in Riga. Response rates are included below.

The large differences in the invited and returned numbers between Meyer and CCUH is assumed to be primarily a consequence of different enrolment approaches, as specified in the methods section, with CCUH taking an opt-out approach and Meyer an opt-in approach (for more detail, see Table 3).

**Table 3:** Number of discharged patients contacted and responses, per hospital

	Invited	Returned	Returned response rate	Returned fully completed	Fully complete response rate
Meyer	2834	1661	58.61%	1080	38.11%
CCUH	14,568	6585	45.20%	4732	32.48%

For Meyer, the majority of respondents are mothers alone (65%), followed by fathers alone (16%) and parents alone (9%). The earlier majority of respondents are aged between 39 and 45 years. Reported citizenship was Italian for (94%) of females and (96%) of males. High school diplomas are held by (38%) of female and (45%) of male respondents, with (47%) and (29%) respectively holding a university degree.

In Riga, the majority of respondents are mothers alone (80%), followed by mothers together with the adolescents (7%) and fathers alone (6%). The earlier majority of respondents are aged between 32 and 38 years. (97%) of females and (92%) of males are Latvians and both female and male respondents mainly have a university degree (71%; 58%) and a high school diploma (24%; 33%).

We used a Pearson chi-squared analysis to investigate differences among socio-demographic variables between the two groups. For age, type of respondents, citizenship, education and employment status, the differences between Italian and Latvian respondents were small but significant (*P* = 0.000), as expected given the sample size. Socio-demographic details are included in Additional file 1.

### Fisher’s exact analysis

In presenting the results below, it is important to consider the analytical approach and participants’ characteristics described above: the significant majority of responses are provided by a carer, with only a small minority provided by children or adolescents together with a carer, and direct responses by adolescents representing only a few percentage points (see above and Additional file 1 for details). It is not possible to determine the degree of involvement of the child or adolescent when responding together with a parent. Given the very low numbers of direct responses from children and adolescents, and the broader focus of this study, all results are reported together. As such, unless explicitly stated otherwise, all reporting of experience includes data that are directly reported by children or adolescents as well as that reported in conjunction with a caregiver, and – as in the vast majority of cases – that are proxy reported by a carer.

In general, the mean scores for domains within child and adolescent experience, caregiver experience, and ward admission and comfort are very closely matched between Meyer and CCUH, though within these means there is some divergence; in some cases, scores from Meyer hospital are more skewed towards both the highest and lowest ends of the spectrum, while CCUH is more grouped around the mean. This is the case for experience of children and adolescents in being treated with respect and dignity by nurses and by other ward staff, and for being involved in decisions about their care as much as they would like; for caregiver experience, this effect is observed for privacy, involvement, information, and team work. In the ward comfort domain, the effect is seen for scores relating to food, and ward entertainment. The result is that the hospitals from both countries provide almost the same mean scores.

The exception is for the discharge phase, where CCUH has higher mean scores than Meyer. The following paragraphs set out these results in full.

### Experience of children and adolescents

As can be seen in the table below, in the ‘patient-provider communication’ and ‘respect and dignity’ dimensions, patients at Meyer report a slightly more positive experience than at CCUH.

Questions without significant differences (*p*-value> 0.05) were those related to being encouraged to ask questions by nurses, being treated in a right way according to age, being treated with respect and dignity by doctors (*p* < 0.100), supported in facing fears and anxiety, and helped for pain management. Other questions showed differences with *p*-value< 0.05 or smaller.

Table 4 shows the percentage of respondents indicating the lowest possible or highest possible experience scores for each question (% of respondents) in the patient experience domain.

**Table 4:** Lowest and highest scores by dimension in the patient experience domain (%)

		CCUH		Meyer		
Dimension	Item	Lowest	Highest	Lowest	Highest	Fisher’s exact
Patient-provider communication	Listened carefully by doctors	0.50	61.92	1.46	62.93	0.001
Patient-provider communication	Listened carefully by nurses	0.45	59.64	0.00	66.59	0.017
Patient-provider communication	Given clear explanations by doctors	1.39	54.73	2.20	63.17	0.002
Patient-provider communication	Given clear explanations by nurses	2.03	50.62	1.71	63.90	0.000
Patient-provider communication	Encouraged to ask by doctors	8.33	33.76	11.95	36.34	0.045
Patient-provider communication	Encouraged to ask by nurses	9.97	29.45	11.22	32.93	0.340
Patient-provider communication	Treated in an age proper way	0.73	59.36	0.93	60.71	0.114
Respect and dignity	Respect and dignity by doctors	0.32	81.56	0.00	85.46	0.073
Respect and dignity	Respect and dignity by nurses	0.14	73.76	0.18	82.31	0.000
Respect and dignity	Respect and dignity by other ward staff	0.61	65.54	0.71	79.64	0.000
Fears and anxiety	Fears and anxiety	1.59	55.46	1.84	57.58	0.619
Pain	Pain	0.64	73.97	0.41	75.16	0.762
Involvement	Involvement	6.08	43.92	4.24	58.47	0.044

The different distributions between the two is well illustrated by questions asking about being given clear explanations: in Meyer, it emerged that children and adolescents were always given understandable explanations by doctors and nurses 63 and 64% of the time, and that they were usually understandable 26% of the time. In Riga, explanations were always clear 55 and 51% of the time, and usually clear 35 and 37% of the time.

### Caregiver’s experience

As shown in the table below, caregivers at Meyer hospital reported slightly better experience for most items within this domain: having their fears and anxieties addressed, being involved in choices, kept informed, and ward staff working together. Differences in mean scores are very small, with better reported experience in Meyer driven by higher scores on the always responses, alongside lower scores on the usually domain. Since mean differences are very small, full results are reported only in the appendix. Caregivers at CCUH reported better experience for being given privacy to discuss the care of the patient and being given clear answers by doctors, driven by higher scores on both always and usually answers. All show low *p*-values (p-value = 0.000). There were no significant differences between the hospitals on the questions of having their fears and anxieties addressed and being given clear answers by nurses (p-value> 0.05).

Table 5 shows the percentage of respondents indicating the best possible or worst possible experience for each question (% of respondents) in the caregiver experience domain.

**Table 5:** Lowest and highest scores by dimension in the caregiver experience domain (%)

		CCUH		Meyer		
Dimension	Item	Lowest	Highest	Lowest	Highest	Fisher’s exact
Fears and anxiety	Fears and anxiety	2.36	54.36	2.24	56.91	0.422
Privacy	Privacy	1.62	57.84	6.17	51.37	0.000
Involvement	Involvement	3.57	52.27	2.06	61.06	0.000
Caregiver-provider communication	Doctor-caregiver communication	0.37	76.56	0.35	75.13	0.000
Caregiver-provider communication	Nurse-caregiver communication	0.39	69.95	0.62	68.08	0.365
Information	Information	1.67	47.96	1.63	65.95	0.000
Team work	Team work	0.45	51.50	1.47	55.54	0.000

**Fig. 1:**
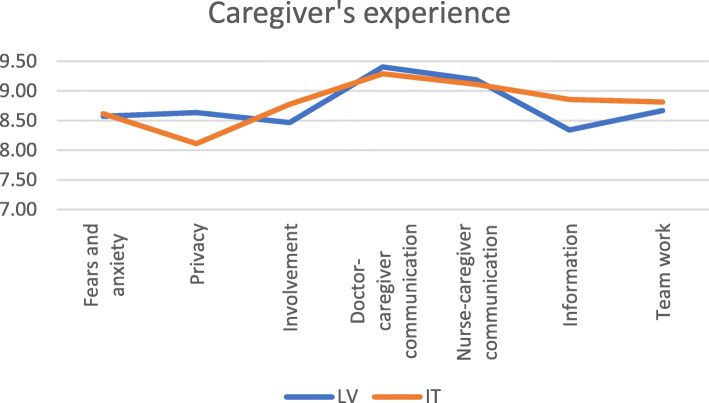
Mean caregiver experience scores

In Fig. 1, to illustrate the variability among dimensions within this domain, we represented the means of the answers provided for each question item composing this domain. Response-scales vary across and within sections of the questionnaire so all responses were normalized to a 1–10 scale to make results comparable.

### Ward admission and comfort

**Fig. 2:**
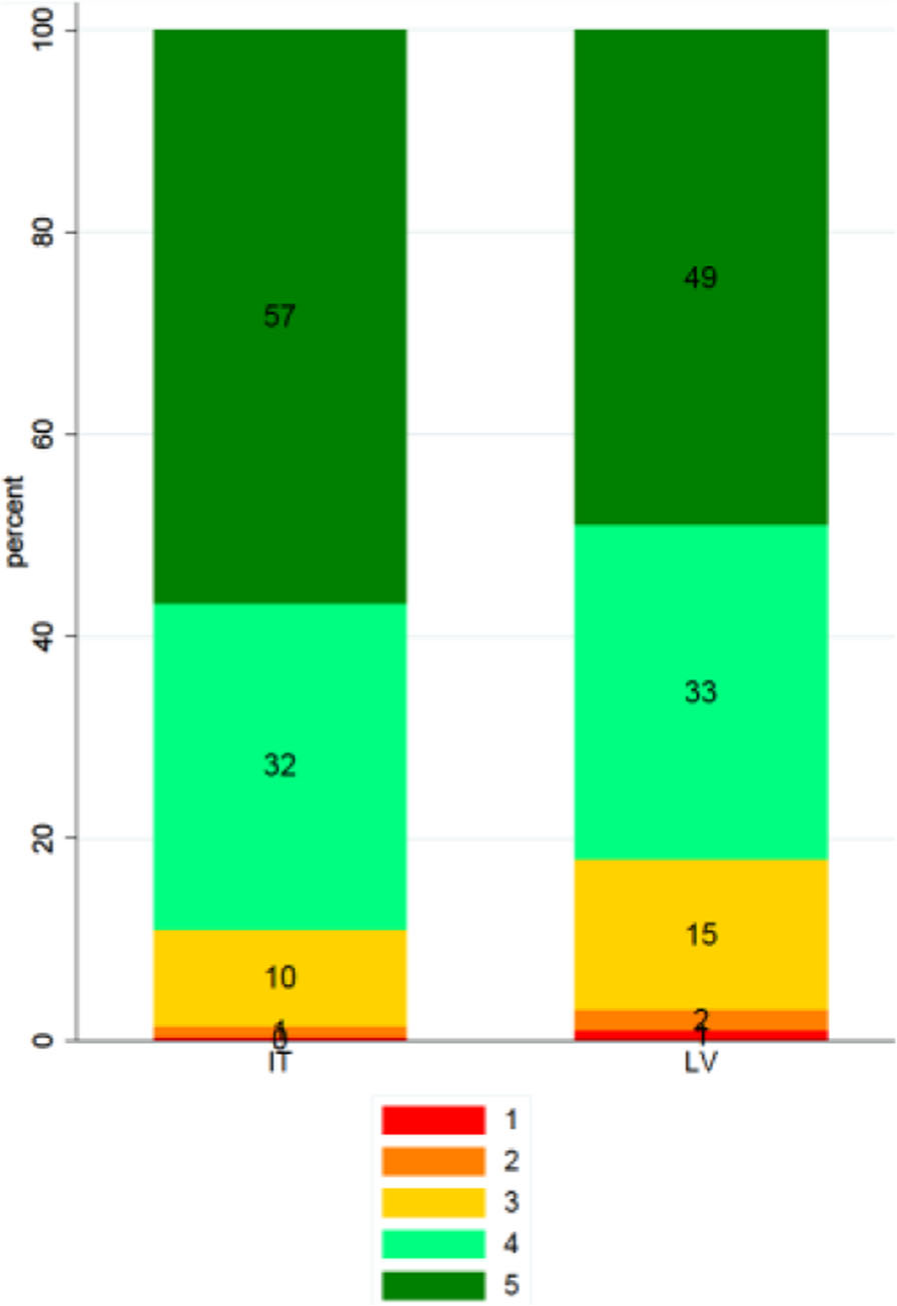
Answers provided about the kindness and courtesy at the admission dimension (%)

Regarding the dimension of ‘kindness and courtesy at the admission’, illustrated in Fig. 2, respondents from Meyer scored higher than respondents from CCUH, with *p*-value = 0.000.

In Fig. 2, the scores correspond to the 5-level response scale of the question regarding being treated with kindness and courtesy at the time of admission, ranging from the lowest score (1 = red) corresponding to “Not at all” to the highest score (5 = dark green) corresponding to “Very much”.

Additionally, respondents from CCUH reported better scores for lack of noise and cleanliness, while those from Meyer scored more highly on satisfying food, and appropriate entertainment. All *p*-values are low (p-value = 0.000).

Table 6 shows the percentage of respondents indicating the best possible or worst possible experience for each question (% of respondents) in the ward comfort domain.

**Table 6:** Lowest and highest scores by dimension in the ward comfort domain (%)

	CCUH		Meyer		
Dimension	Lowest	Highest	Lowest	Highest	Fisher’s exact
Silence	1.56	54.95	1.09	33.19	0.000
Cleanliness	0.47	55.73	0.50	28.34	0.000
Food catering service	5.46	28.10	3.73	44.08	0.000
Entertainment	24.75	53.43	14.80	64.72	0.000

**Fig. 3:**
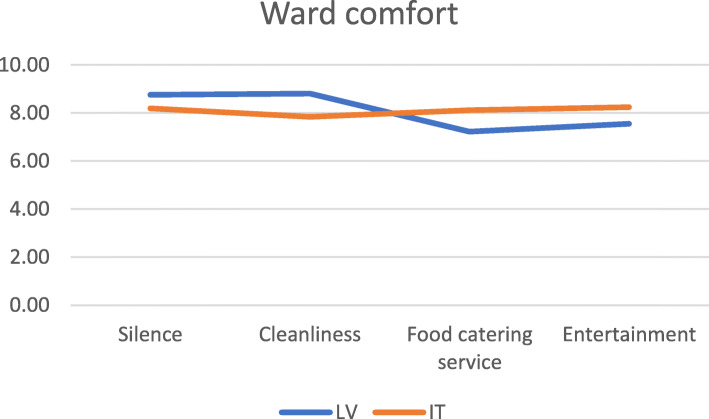
Mean ward comfort scores

Again, in Fig. 3, in order to show variability among dimensions within this domain, we represented the means of the answers provided for each question item composing this domain, after adjusting them from different response scales to a 1–10 scale.

### Discharge phase

The discharge phase showed the greatest divergence of mean scores, and overall higher scores for CCUH, unlike for the domains described above.

Compared with Meyer, respondents at CCUH report a slightly more positive experience with respect to a number of dimensions in this domain, namely:

- if a provider asked the caregiver if she had concerns about whether the child or adolescent was ready to leave (49%; 73%) with *p*-value = 0.000
- if a provider talked with the caregiver as much as she wanted about how to care for the child’s or adolescent’s health after leaving the hospital (79%; 86%) with p-value = 0.000
- if a provider explained in a way that was easy to understand when the child or adolescent could return to his or her daily activities (73%; 74%) with *p*-value< 0.01
- if a provider explained in a way that was easy to understand how the child or adolescent should take new medicines after leaving the hospital (87%; 92%) with *p*-value< 0.05
- if a provider explained in a way that was easy to understand what symptoms or health problems to look out for after leaving the hospital (73%; 74%) although differences are not statistically significant
- if the information written in the discharge letter was clear (44%; 46%) with *p*-value< 0.01
- if the information written in the received materials (except the discharge letter) was clear (43%; 48%) with p-value = 0.000

On the other hand, respondents at Meyer report a slightly more positive experience than at CCUH in this domain regarding:

- if a provider explained in a way that was easy to understand about possible side effects of the new medicines (49%; 41%) with p-value< 0.01
- if the specific training received in order to personally take care of the child or adolescent once back home was useful (61%; 52%) with *p*-value = 0.000

Table 7 shows the percentage of respondents indicating the best possible or worst possible experience for each question (% of respondents) in the discharge phase domain.

**Table 7:** Lowest and highest scores by dimension in the discharge phase domain (%)

		CCUH		Meyer		
Dimension	Item	Lowest	Highest	Lowest	Highest	Fisher’s exact
Information	Ready to be discharged	10.97	72.88	30.60	49.08	0.000
Information	Managing care at home	3.31	85.99	4.93	78.51	0.000
Information	Back to daily routine	10.44	74.36	8.11	73.16	0.002
Information	New drugs administration	1.46	91.75	2.93	88.64	0.016
Information	New drugs side effects	41.03	40.50	36.14	49.05	0.001
Information	Symptoms to monitor	9.51	74.22	11.27	73.28	0.244
Information	Discharge letter	0.46	45.65	0.66	43.64	0.001
Information	Other written materials	0.18	48.37	0.19	42.78	0.000
Training	Training	0.35	51.77	0.24	60.99	0.000

### Overall evaluation

The majority of respondents, either at Meyer or at CCUH, reported that the care received by the child or adolescent in the ward was very good (63%; 59%), while a significant percentage of these two groups reported that it was good (31%; 33%). Respondents at Meyer, again, report a slightly more positive experience than at CCUH with p-value = 0.000.

Finally, the majority of respondents, either at Meyer or at CCUH, reported the child’s or adolescent’s current health status as good (34%; 30%), with a significant percentage of these two groups reporting that it is very good (19%; 13%). The latter cannot be considered as an indicator of quality of care as it is not possible to control for initial health status or case-mix of respondents.

Full results of Fisher’s exact analysis, with significance values, are included in Additional file 1.

### Anecdotal evidence from open comments

Analysis of comments received at Meyer shows a high proportion of children and adolescents or carers choose to provide narrative comments, with a majority of positive comments. Questions addressed directly to children and adolescents were often answered by them in the first person, although it is not possible to verify to what extent these responses are autonomously provided by children and adolescents, or mediated by carers. Other items are expected to have been completed by the caregiver. Analysis of comments using text analysis software identified the most frequently used terms in storytelling as kindness, availability and professionalism.

Most narrative comments at CCUH are provided by parents to thank staff for caring for their children. Of more than 7000 comments - 30% were negative or contained some suggestions for improvement. Most recommendations were about improving the environment and food quality. Most of positive comments were about kind and positive attitudes and clear communication.

From the ward admission stage, typical comments show that it is important to provide children, adolescents and their caregivers with information on what they should expect from the hospital stay, for example which test and examinations will be performed and their expected waiting times. Another relevant fairly common concern regards room assignment and availability of beds, in particular for scheduled admissions. On the other hand, other comments note that admission is often quick and comfortable, and ward personnel are kind and warmly welcoming.

In the hospitalization experience of children and adolescents, some points relate to difficulties in the interpersonal relationships between the child or adolescent and the hospital staff. Although ward staff were usually reported as polite, there are sometimes difficulties expressed in communication with health workers. However, generally a very positive attitude emerges from the child, with hospital staff perceived as paying lots of attention to children and adolescents, making their overall experience of hospitalization positive.

Common criticisms from the adult’s perspective relate to interpersonal relationships towards children, adolescents and their caregivers, notably insensitivity, challenges in getting desired information, and the impression of a lack of information exchange between hospital staff. This last observation can be usefully compared with the questionnaire question “How would you rate the ability of the ward medical and nursing staff to work together?”, for which responses are largely positive ​​(very good 55.10% and good 34.33%). However, for the share of caregivers who found it deficient, this aspect was evidently relevant to them, as highlighted by several comments in the storytelling (example shown in Additional file 1).

A significant number of respondents were critical about the ward environment, especially regarding the quality and efficiency of food catering service during the hospitalization. Nevertheless, generally, there is a positive opinion of many wards where children and adolescents are hospitalised, especially in terms of cleanliness, equipment and comfort.

Finally, when evaluating the hospital staff, comments are generally very positive, with respondents expressing gratitude to those who took care of them.

The themes emerging are as expected based on what patients are known to value during their hospitalisation experience. Additionally, these insights show that open comments can be a tool to value and motivate personnel; responses clearly illustrate that each health worker can make a difference in the child’s and adolescent’s experience, and that the respondents are able to recognize their important role in service provision.

Comments can also be used to identify potential improvements; it clearly emerges that ward staff could sometimes better understand the impact their behaviours have on children and adolescents, for example in choosing the right time and place to have breaks and personal interactions, or in giving the appearance of detachment from children, adolescents and carers. The different absolute numbers of comments for Meyer and CCUH correspond to the difference in the numbers of returned questionnaires (see Table 8). A sample of the comments is presented in Additional file 1, selected to represent the range of expressed views.

**Table 8:** Number of free text comments received

	Emergency department	Ward admission	Hospitalization experience of children and adolescents	Caregiver’s point of view	Ward environment	Staff evaluation	Total
Meyer	n/a	733	290	486	432	552	2493
CCUH	1294	2206	883	1439	1440	2010	9272

## Discussion

The model of continuous digital collection of paediatric PREMs described above appears to be a feasible option for collecting paediatric PREMs at scale (see also [18]). The large population reached in both countries indicates that the interface between survey platform and hospital data systems is an effective method of survey administration, while high response rates suggest that the electronic collection model is acceptable and convenient for respondents. The difference between survey responses and fully completed survey responses of around a third in both hospitals suggests that the current questionnaire may be overly long, discouraging full completion among some respondents – although this should be considered with reference to the different enrolment methods.

The collection of qualitative comments is important in sustaining front line staff interest, and increases the insight possible from data collection by providing greater richness of information – though how far this applies depends on the developmental status of respondents and may not always hold true for younger children. In future, approaches such as text mining and natural language processing could facilitate the use of qualitative comments in identifying improvement opportunities and potentially to derive new insights to improve the experience of children and adolescents through analysis of large numbers of qualitative responses. The data platform and hosting provide a simple way for hospital teams to access data at different levels of granularity, as well as enabling secondary analysis and application of advanced analytical techniques.

Insights gathered from hospital staff in management of the model report a differentiated use of answers according to their nature. Hospital management use negative answers to identify problems in care delivery and intervene promptly in order to resolve them. Positive answers are used to motivate all categories of hospital staff, diffuse positive behaviours within the organization and spread good practices through a Learning from Excellence (LfE) model [19].

The data indicate that both hospitals provide good experience for children and adolescents - as reported by caregivers - with little variation in performance across experience domains. There is a notable extent of agreement between respondents in the two countries, despite attending hospitals with different cultures and systems, having different cultural backgrounds, different levels of wealth in the European context, and different languages. Further exploration of the role of cultural differences in reported patient experience would enable a more nuanced interpretation of the distributions of scores. However, these differences must be set in the context of some similarities: Latvia and Italy are both European countries and members of the European Union, both participating organisations are specialist paediatric hospitals and based in cities, and both are regarded as high performing and are members of the European Children’s Hospitals Organisation (CCUH joined ECHO during the period of this study).

The qualitative comments suggest that the similar results are because there are broadly shared preferences among children and adolescents in the two countries, and that both hospitals in this study are similarly effective at meeting expectations – or at least, as reported by carers.

The major limitation of the model is that the questionnaire is not entirely framed for and offered exclusively to children and adolescents, given that the purpose is to measure their experiences of hospitalization. While this is the approach for most surveys of children and adolescents, and parental reporting is a good source of information for younger children especially [20], there is evidence that surveys can be developed which are feasible and acceptable for children over the age of 8 to answer directly, and that their responses provide additional insights to those available from parental responses alone [21].

The survey described here was based on an existing adult experience survey, adapted such that it can be filled in by parents or adolescents. On the one hand, this can enable comparison of items between paediatric and non-paediatric hospitals, supporting evaluation and learning, and enables a single survey to be used rather than multiple surveys for different age groups [22]; on the other, the survey may lack relevant items for youth-friendly healthcare [23], may not be best configured to encourage clarity and minimise fatigue among respondents, and is not appropriate for younger children to fill in directly.

This feature may have encouraged the situation that, although adolescents were invited to participate in the survey autonomously, parents responding alone form the significant majority of total respondents. More specifically, parents responding with a child or adolescent represent a significant minority, while adolescents alone form only a minimal proportion of respondents (3%). A survey which could be filled in autonomously by a wider selection of children and adolescents would require significant development work (or validation of a pre-existing survey for these settings), but could capture additional insights into the experiences of children and adolescents in participating hospitals. Further research in CCUH and Meyer hospitals would be of value to determine how the survey could be developed to make it appropriate for younger children to autonomously complete. Additionally, there could be value in developing separate developmentally-appropriate versions of the questionnaire for children, adolescents and carers, rather than embedding all questions in a questionnaire mostly answered by caregivers. Such an adjustment process would apply to both the closed-ended and open-ended questions.

Another limitation is that we do not know the demographic details of all those who were invited to participate in the survey, and therefore cannot determine how proportionate the number of responding adolescents is to all those invited, and to the total eligible patient population. This issue further complicates the analysis of results and makes more unclear how far this “proxy reporting by parents” effect is driven by the survey enrolment or administration model, by the survey itself, or by something else. Including a function in the PREMs administration system to collect the demographic characteristics of all those who were invited to participate in the survey could help address this.

Finally, the impossibility of case mix comparisons or of adjusting for initial health status may be considered a limitation in our analysis, although evidence on the link between patient experience and severity of disease is inconclusive.

There was no formal pilot period in developing the PREMs model, which helps explain a number of the limitations described here. This was due to the need to align with timelines determined by the wider Latvian HSPA development, supported by the structural reform programme, which necessitated a shorter period of development and testing than could include e.g. development and testing of bespoke surveys for different developmental levels. The priority for the initial phase of operation was to implement the technologies and processes which enable the collection of survey data at scale. Operating the PREMs survey within the two hospitals has served as the proof-of-concept and establishment of protocols; subsequent in-country ownership and leadership of PREMs collection could provide the opportunity to resolve the most important of the limitations described above. As well as potential adjustments to the recruitment process or to the survey itself, this might additionally include greater ability to categorise responses by disease group and to provide an additional level of insight than the currently available ward level. The benefits available from benchmarking suggest any changes should be implemented in both Meyer and CCUH hospitals to ensure ongoing comparability.

## Conclusions

In this study we demonstrate the possibility of establishing an international benchmarking system for paediatric PREMs in two European countries, and compare survey responses between Florence and Riga.

The features of this PREMs collection and management model are relevant for researchers and policymakers as well as for front line staff and managers. The OECD [9] criteria to determine the scope of conditions and sectors for patient reported data collection are: actionability, relevance, cost of implementation, availability of measures and feasibility of collection. The model implemented in Meyer and CCUH is well suited to all these criteria: a high response rate, cheap and efficient contact to and data collection from a wide population, and simple, efficient recording and collating of responses in real time. While there are limitations which could be addressed – notably the current reliance on proxy carer reporting of child and adolescent experience - the model enables a large, longitudinal dataset to be collected over time, providing greater use as a management tool and for the purposes of benchmarking. This model could be effective at meeting the OECD’s criteria, help deliver the call for expansion of PREM data collection, and enable a focus on international benchmarking.

The insights and value possible from benchmarking in this way would increase as the number of comparators increases; other countries or providers seeking to commence or derive greater benefit from PREM data collection may wish to explore adopting this or comparable models of data collection. The continuous data collection and large eligible population (as opposed to periodic sampling methods) make this model particularly useful for benchmarking, notably through allowing real time or retrospective investigation of discrete events and evaluations of initiatives by bringing in scope additional analytical techniques, such as interrupted time series analysis.

Accordingly, further research of practical benefit should include the wider benchmarking of CCUH and Meyer with other specialist paediatric hospitals and other paediatric respondents in a general hospital, and exploring the drivers of variation in these scores. From this, new insights may emerge regarding the most effective ways to deliver a positive experience for children and adolescents, as well as approaches which help deliver important domains of patient experience – whether associated with overall satisfaction or not. It is our hope that such research will become increasingly possible and widespread as other countries and providers heed the call of the OECD and adopt PREMs models which enable international benchmarking, particularly those providing large longitudinal data sets as described here.

In the longer term, a valuable focus would be to extend this or similar digital, continuous systems for paediatric PREMs collection and analysis to other national and international hospitals. In doing so, a larger network of healthcare organizations sharing a common benchmarking platform could be established to support continuous performance improvement.

Moreover, future research should also investigate how the PREMs system was implemented in each healthcare system, and to understand how this affects participation rates and staff support for the programme. Additional research of value would evaluate the use of the data return web platform as a management tool to improve the quality of care.

## Supplementary Information

**Additional file 1.**

**Additional file 2.**

## Data Availability

The datasets analysed are available from the corresponding author upon reasonable request.

## Abbreviations

PREMs: Patient Reported Experience Measures
Meyer: Meyer Children’s University Hospital
CCUH: Children’s Clinical University Hospital
CYP: Children and Young People
OECD: Organisation for Economic Co-operation and Development
HSPA: Health System Performance Assessment
SRSS: Structural Reform Support Service
API: Application Programming Interface
CAHPS: Consumer Assessment of Healthcare Providers and Systems
MeS Lab: Management and Healthcare Laboratory
LfE: Learning from Excellence
ECHO: European Children’s Hospitals Organisation
