# Supplementary material for: Benchmarking experience to improve paediatric healthcare: listening to the voices of families from two European Children’s University Hospitals
Source: BMC Health Serv Res. 2021 Jan 27;21:93. doi: 10.1186/s12913-021-06094-z (PMC7839229; doi:10.1186/s12913-021-06094-z)
Supplement: Supplementary file 1 — Additional file 1. [file 12913_2021_6094_MOESM1_ESM.docx]

Annex A

Respondents grouped by type of respondent (%)

| Type of respondent | CCUH | Meyer | P-value |
| --- | --- | --- | --- |
| Mother | 80,38 | 65,05 | 0.000 |
| Mother together with the adolescent (14-17 years) | 6,55 | 5,02 | 0.000 |
| Father | 5,94 | 16,30 | 0.000 |
| Father together with the adolescent (14-17 years) | 0,45 | 1,51 | 0.000 |
| Parents | 2,49 | 8,95 | 0.000 |
| Parents together with the adolescent (14-17 years) | 0,33 | 0,50 | 0.000 |
| Custodial parent | 0,43 | 0,00 | 0.000 |
| Custodial parent together with the adolescent (14-17 years) | 0,14 | 0,08 | 0.000 |
| Legal guardian | 0,60 | 0,08 | 0.000 |
| Legal guardian together with the adolescent (14-17 years) | 0,04 | 0,00 | 0.000 |
| Adolescent (14-17 years) alone | 2,65 | 2,51 | 0.000 |

|  | CCUH | Meyer | CCUH | Meyer | P-value |
| --- | --- | --- | --- | --- | --- |
|  | Female | | Male | |  |
| Respondents by age group (%) | | | | | |
| <18 years | 0,27 | 0,00 | 0,12 | 0,00 | 0.000 |
| 18-24 | 2,94 | 0,68 | 1,00 | 0,17 | 0.000 |
| 25-31 | 22,46 | 7,36 | 16,54 | 3,90 | 0.000 |
| 32-38 | 39,03 | 29,70 | 34,27 | 18,70 | 0.000 |
| 39-45 | 25,31 | 36,80 | 27,69 | 36,71 | 0.000 |
| 46-50 | 6,56 | 17,51 | 9,70 | 21,99 | 0.000 |
| 51-55 | 2,49 | 6,09 | 4,76 | 11,60 | 0.000 |
| 56-60 | 0,45 | 1,69 | 1,50 | 5,02 | 0.000 |
| 61-65 | 0,33 | 0,17 | 0,58 | 1,21 | 0.000 |
| >65 years | 0,06 | 0,00 | 0,17 | 0,69 | 0.000 |
| Respondents by citizenship (%) | | | | | |
| Latvian | 96,69 | . | 91,86 | . | 0.000 |
| Non-Latvian | 3,31 | . | 8,14 | . | 0.000 |
| Italian | . | 93,74 | . | 95,55 | 0.000 |
| Non-Italian | . | 6,26 | . | 4,45 | 0.000 |
| Respondents by employment status (%) | | | | | |
| Employed | 77,78 | 67,15 | 94,82 | 91,27 | 0.000 |
| Unemployed | 22,22 | 32,85 | 5,18 | 8,73 | 0.000 |
| Respondents by level of education (%) | | | | | |
| No title | 0,29 | 0,34 | 0,97 | 0,26 | 0.000 |
| Primary school | NA | 0,59 | NA | 1,39 | 0.000 |
| Middle school | 4,96 | 14,12 | 8,57 | 23,81 | 0.000 |
| High school | 23,53 | 38,21 | 32,59 | 45,19 | 0.000 |
| Bachelor degree | 53,10 | 37,28 | 46,63 | 23,72 | 0.000 |
| Master degree or PhD | 18,12 | 9,47 | 11,23 | 5,63 | 0.000 |
| University degree | 71,22 | 46,75 | 57,86 | 29,35 | 0.000 |

Annex B

| Domain | Dimension | CCUH | | | | | Meyer | | | | |  |
| --- | --- | --- | --- | --- | --- | --- | --- | --- | --- | --- | --- | --- |
|  |  | Never | Rarely | Sometimes | Often | Always | Never | Rarely | Sometimes | Often | Always | Fisher's exact |
| Patient's experience | Respect and dignity by doctors | 0.32 | 0.67 | 2.38 | 15.06 | 81.56 | 0.00 | 0.70 | 2.80 | 11.03 | 85.46 | 0.073 |
| Patient's experience | Respect and dignity by nurses | 0.14 | 0.99 | 4.22 | 20.89 | 73.76 | 0.18 | 1.05 | 3.50 | 12.96 | 82.31 | 0.000 |
| Patient's experience | Respect and dignity by other ward staff | 0.61 | 2.42 | 8.05 | 23.39 | 65.54 | 0.71 | 1.07 | 4.11 | 14.46 | 79.64 | 0.000 |
| Patient's experience | Fears and anxiety | 1.59 | 3.09 | 10.21 | 29.65 | 55.46 | 1.84 | 3.28 | 7.99 | 29.30 | 57.58 | 0.619 |
| Patient's experience | Pain | 0.64 | 1.19 | 5.10 | 19.10 | 73.97 | 0.41 | 0.62 | 5.80 | 18.01 | 75.16 | 0.762 |
| Patient's experience | Involvement | 6.08 | 4.59 | 14.52 | 30.89 | 43.92 | 4.24 | 4.24 | 7.63 | 25.42 | 58.47 | 0.044 |
| Caregiver's experience | Fears and anxiety | 2.36 | 3.36 | 11.87 | 28.05 | 54.36 | 2.24 | 3.56 | 12.30 | 25.00 | 56.91 | 0.422 |
| Caregiver's experience | Involvement | 3.57 | 3.84 | 10.61 | 29.71 | 52.27 | 2.06 | 4.03 | 8.23 | 24.61 | 61.06 | 0.000 |
| Caregiver's experience | Doctor-caregiver communication | 0.37 | 0.95 | 3.45 | 18.68 | 76.56 | 0.35 | 1.93 | 5.78 | 16.81 | 75.13 | 0.000 |
| Caregiver's experience | Nurse-caregiver communication | 0.39 | 1.45 | 6.38 | 21.82 | 69.95 | 0.62 | 1.41 | 7.76 | 22.13 | 68.08 | 0.365 |
| Ward admission and comfort | Food catering service | 5.46 | 13.87 | 22.84 | 29.73 | 28.10 | 3.73 | 5.83 | 15.66 | 30.69 | 44.08 | 0.000 |
|  |  |  |  |  |  |  |  |  |  |  |  |  |
| Domain | Dimension | CCUH | | | | | Meyer | | | | |  |
|  |  | Not at all | A little bit | Enough | Much | Very much | Not at all | A little bit | Enough | Much | Very much | Fisher's exact |
| Ward admission and comfort | Kindness and courtesy at the admission | 1.09 | 2.04 | 14.90 | 33.05 | 48.92 | 0.42 | 1.09 | 9.62 | 32.27 | 56.61 | 0.000 |
| Ward admission and comfort | Silence | 1.56 | 3.76 | 5.22 | 34.51 | 54.95 | 1.09 | 3.51 | 13.46 | 48.75 | 33.19 | 0.000 |
| Ward admission and comfort | Cleanliness | 0.47 | 1.19 | 11.63 | 30.98 | 55.73 | 0.50 | 4.68 | 25.50 | 40.97 | 28.34 | 0.000 |
| Discharge phase | Discharge letter | 0.46 | 2.63 | 15.09 | 36.17 | 45.65 | 0.66 | 0.77 | 13.93 | 41.01 | 43.64 | 0.001 |
| Discharge phase | Other written materials | 0.18 | 1.81 | 15.55 | 34.09 | 48.37 | 0.19 | 0.56 | 12.76 | 43.71 | 42.78 | 0.000 |
| Discharge phase | Training | 0.35 | 1.47 | 16.54 | 29.87 | 51.77 | 0.24 | 1.18 | 21.51 | 16.08 | 60.99 | 0.000 |
|  |  |  |  |  |  |  |  |  |  |  |  |  |
| Domain | Dimension | CCUH | | | | | Meyer | | | | |  |
|  |  | Very poor | Poor | Sufficient | Good | Very good | Very poor | Poor | Sufficient | Good | Very good | Fisher's exact |
| Caregiver's experience | Team work | 0.45 | 1.73 | 13.18 | 33.13 | 51.50 | 1.47 | 2.08 | 6.31 | 34.60 | 55.54 | 0.000 |
| Overall evaluation | Perceived care quality | 0.21 | 0.68 | 7.74 | 32.78 | 58.59 | 0.75 | 1.17 | 3.51 | 31.44 | 63.13 | 0.000 |
|  |  |  |  |  |  |  |  |  |  |  |  |  |
| Domain | Dimension | CCUH | | | | | Meyer | | | | |  |
|  |  | Poor | Fair | Good | Very good | Excellent | Poor | Fair | Good | Very good | Excellent | Fisher's exact |
| Overall evaluation | Patient health status | 2.05 | 18.86 | 36.05 | 29.95 | 13.09 | 2.51 | 14.21 | 30.85 | 33.86 | 18.56 | 0.000 |

| Domain | Dimension | CCUH | | | | Meyer | | | |  |
| --- | --- | --- | --- | --- | --- | --- | --- | --- | --- | --- |
|  |  | Never | Sometimes | Usually | Always | Never | Sometimes | Usually | Always | Fisher's exact |
| Patient's experience | Listened carefully by doctors | 0.50 | 6.35 | 31.23 | 61.92 | 1.46 | 10.49 | 25.12 | 62.93 | 0.001 |
| Patient's experience | Listened carefully by nurses | 0.45 | 4.86 | 35.05 | 59.64 | 0.00 | 5.61 | 27.80 | 66.59 | 0.017 |
| Patient's experience | Given clear explanations by doctors | 1.39 | 8.48 | 35.40 | 54.73 | 2.20 | 8.29 | 26.34 | 63.17 | 0.002 |
| Patient's experience | Given clear explanations by nurses | 2.03 | 10.61 | 36.74 | 50.62 | 1.71 | 8.29 | 26.10 | 63.90 | 0.000 |
| Patient's experience | Encouraged to ask by doctors | 8.33 | 24.24 | 33.66 | 33.76 | 11.95 | 22.68 | 29.02 | 36.34 | 0.045 |
| Patient's experience | Encouraged to ask by nurses | 9.97 | 26.28 | 34.31 | 29.45 | 11.22 | 23.41 | 32.44 | 32.93 | 0.340 |
| Patient's experience | Treated in an age proper way | 0.73 | 6.98 | 32.93 | 59.36 | 0.93 | 9.31 | 29.05 | 60.71 | 0.114 |
| Caregiver's experience | Privacy | 1.62 | 9.28 | 31.25 | 57.84 | 6.17 | 14.58 | 27.87 | 51.37 | 0.000 |
| Caregiver's experience | Information | 1.67 | 10.97 | 39.40 | 47.96 | 1.63 | 8.40 | 24.01 | 65.95 | 0.000 |

| Domain | Dimension | CCUH | | | Meyer | | |  |
| --- | --- | --- | --- | --- | --- | --- | --- | --- |
|  |  | No | Yes, somewhat | Yes, definitely | No | Yes, somewhat | Yes, definitely | Fisher's exact |
| Ward admission and comfort | Entertainment | 24.75 | 21.82 | 53.43 | 14.80 | 20.48 | 64.72 | 0.000 |
| Discharge phase | Ready to be discharged | 10.97 | 16.15 | 72.88 | 30.60 | 20.32 | 49.08 | 0.000 |
| Discharge phase | Managing care at home | 3.31 | 10.70 | 85.99 | 4.93 | 16.56 | 78.51 | 0.000 |
| Discharge phase | Back to daily routine | 10.44 | 15.20 | 74.36 | 8.11 | 18.73 | 73.16 | 0.002 |
| Discharge phase | New drugs administration | 1.46 | 6.79 | 91.75 | 2.93 | 8.43 | 88.64 | 0.016 |
| Discharge phase | New drugs side effects | 41.03 | 18.48 | 40.50 | 36.14 | 14.80 | 49.05 | 0.001 |
| Discharge phase | Symptoms to monitor | 9.51 | 16.28 | 74.22 | 11.27 | 15.45 | 73.28 | 0.244 |

Annex C

Hospital and ward admission

“When we were told that there were no beds, even if it was a scheduled hospitalization, I don't understand how you can leave a child 3 hours waiting to be hospitalized, as everything was planned. An absolutely negative experience.” (Comment from Meyer)

“We were located in the room with the patient who had virus although our child was not infectious and had only trauma. We asked for option to have another room. Nurses allowed us to change the room, but it was too late already. My child had the same symptoms as patient in previous room. We could prevent this situation.” (Comment from CCUH)

“Upon arrival there was some confusion at the admission. I expected another type of treatment, knowing the hospital. Afterwards, however they were very good ... in particular the anesthesiologist who came to see my daughter telling her that they were going to the space. She was super calm and couldn't wait.” (Comment from Meyer)

“We were struck by the fact that the doctor who took care of our daughter explained to her from the outset in a simple but very understandable way for her age what she was hospitalized for and what she would go to.” (Comment from Meyer)

“Admission at the ward was quick, well organized and welcoming was kind” (Comment from CCUH)

Paediatric patient’s experience of hospitalization

“I didn’t like that nurse had a bad smell after smoking” (Comment from CCUH)

“They rarely talk with me. I don't like this, but I'm used to it. They helped mom put me in a wheelchair and it made me very happy because she can never do it alone” (Comment from Meyer)

“All kind, very busy, but the problem was that I was hospitalized in a room with the "little ones" and I slept little so I felt more of my pain” (Comment from Meyer)

“I liked being at the hospital because of the staff. All doctors and intern were friendly and were joking. And because of that I didn’t feel scared. Most of the nurses were kind and lovely. I was usually afraid from hospitals, but after this experience I am not afraid anymore.” (Comment from CCUH)

“I was carefully followed, with affection, and made part of the care process.” (Comment from Meyer)

Caregiver’s experience

“The hospital experience has been excellent from a clinical point of view. Nurses and doctors are professional and prepared. I had some difficulties when the child was transferred to the ward: I noticed a lack of communication between the doctors of the two wards. After 3 days, despite my requests for clarification, I learned of the decision to release the child and postpone the intervention until a later date...” (Comment from Meyer)

“The kindness of the staff is an ever-present quality. The environment is beautiful. There should be greater communication between doctors and nurses.” (Comment from Meyer)

“What struck me about this hospitalization was the tranquility with which my son's history was treated. Although the hospitalization was long with a lot of work behind the scenes, I did not feel the weight of the time that passed. The Doctors, the Nurses made us feel as if we were in a large family, very helpful and never nervous about us. All very good...it's a nice group!” (Comment from Meyer)

“Staff was kind, but I didn’t get explanation enough what is wrong with my child. If I don’t ask any questions, nobody would try to explain and would not know anything.” (Comment from CCUH)

“While doctors explained what will happen with my child, what kind of medicaments will be used and procedures will take place, nurse didn’t check my child’s identity, didn’t explained what medicaments is used. When nurse assistants take child to examination, they don’t check the identity or asks for child’s name. They took my child to wrong procedure. This procedure was meant for a girl who was hospitalized in the same room as my child”. (Comment from CCUH)

Hospital environment

“The room in which we were placed was cold and without an internal bathroom, and the keys to the bathroom where we had to go always disappeared.” (Comment from Meyer)

“Heating too high during the day and cold at night. Good opportunity for a parent to sleep in a comfortable cot.” (Comment from Meyer)

“The playroom and library are well-stocked and pleasant to spend time in. The bed for parents is excellent, even compared to other paediatric wards in Tuscany and other Regions. The Meyer remains a vanguard also from the point of view of comfort for children and parents.” (Comment from Meyer)

“We were not satisfied with the food our child received at the hospital. I as a parent had to find and get another food because my child refused to eat it. I am convinced that parents should not think about feeding child at the hospital because they are worried enough about health of the child. Otherwise, everything was ok. Thank you!” (Comment from CCUH)

Hospital staff evaluation

“They were all nice apart at the time of discharge that we had to leave the room and we were parked on the sofa for 4 hours waiting for the discharge without anyone telling us anything and when I tried to ask a nurse how much there was to wait given that my three year old daughter was no longer manageable, the nurse answered me badly...if they work with people and especially with children I think they should be more friendly...” (Comment from Meyer)

“Very professional, tolerant and understanding nurses at the ward 2. Doctors were professional and hearty. Thank you! Many thanks to Pain management doctor for suggestion to use laughing gas when insert catheter.” (Comment from CCUH)

“Some were nice, but others should learn to be more courteous to parents who have young children and need help and be more tender with children too.” (Comment from Meyer)

“Some nurses and nurse assistants were insensitive against patient, despicable against parents, were loud in communication between the staff, when closing the doors while patient is sleeping”. (Comment from CCUH)

“I'd like to report the young surgeon who operated on the child. Before the intervention, she explained in an excellent way, even by drawing it, what she would do. She elucidated the possible consequences in a comprehensive and exhaustive way. Even after the intervention, with kindness and affability, she reassured us on the outcome.” (Comment from Meyer)
