# Supplementary material for: Benchmarking experience to improve paediatric healthcare: listening to the voices of families from two European Children’s University Hospitals
Source: BMC Health Serv Res. 2021 Jan 27;21:93. doi: 10.1186/s12913-021-06094-z (PMC7839229; doi:10.1186/s12913-021-06094-z)
Supplement: Supplementary file 2 — Additional file 2. [file 12913_2021_6094_MOESM2_ESM.docx]

Patient experience questionnaire for hospitalizations of children and adolescents

The questionnaire reported below is the one that is in use in Riga Children’s Clinical University Hospital. The authors reported this version of the questionnaire because it is more exhaustive than the one that is in use at Meyer Children’s University Hospital in Florence. More particularly, the present version investigates the experience of patients and caregivers during the stay in the Emergency Room. A different scale in the two questionnaires characterizes the question regarding the educational level attained by the caregiver, as it reflects the underlying features of the schooling system in place in Latvia and Italy.

Please note that the following question items [13-17, 24, 27, 36, 38-43, 63] are directly from or are somehow inspired to the CAHPS Child Hospital Survey. U.S. Agency for Healthcare Research and Quality, Rockville, MD. Updated 2016.

Available at: https://www.ahrq.gov/cahps/surveys-guidance/hospital/about/child_hp_survey.html (https://www.ahrq.gov/cahps/surveysguidance/hospital/about/child_hp_survey.html)

Accessed March 26, 2018.

1. **Who is filling in the questionnaire?**

- Mother
- Mother together with the adolescent (14-17 years)
- Father
- Father together with the adolescent (14-17 years)
- Parents
- Parents together with the adolescent (14-17 years)
- Custodial parent
- Custodial parent together with the adolescent (14-17 years)
- Legal guardian
- Legal guardian together with the adolescent (14-17 years)
- Adolescent (14-17 years) alone

HOSPITAL ADMISSION

1. **How did the child's/adolescent’s access to this hospital occur?**

- The child/adolescent attended the Emergency Department
- The child/adolescent attended for a previously planned admission
- The child/adolescent was transferred from another hospital

1. **What is the main reason why you chose this hospital?**

*Only answer this question if the following conditions are met:*

*Answer was NOT 'The child/adolescent was transferred from another hospital' at question '2 ' (How did the child's/adolescent’s access to this hospital occur?)*

- I consider it the best hospital for the health problem the child/adolescent has
- The general practitioner of the child/adolescent suggested it to me
- The doctor I have chosen for treating the health problem of the child/adolescent is working there
- A specialist suggested it to me
- My relatives or friends suggested it to me
- It is the closest one to where we live
- I did not choose it, because the child/adolescent had an urgent access through the Emergency Medical Service
- Other

1. **Is the general practitioner of the child/adolescent aware of the child's/adolescent’s hospitalization?**

- Yes, he/she knew before the child/adolescent was hospitalized
- Yes, he/she found out because I contacted him/her during the child/adolescent’s hospitalization
- Yes, he/she found out because the hospital contacted him/her during the child/adolescent’s hospitalization
- Yes, he/she found out because I contacted him/her after the child/adolescent’s hospitalization
- No
- I do not know

1. **With respect to the health problem of the child/adolescent which led to attending the Emergency Department, do you think:**

*Only answer this question if the following conditions are met:*

*Answer was 'The child/adolescent attended the Emergency Department' at question '2' (How did the child's/adolescent’s access to this hospital occur?)*

- The waiting time was shorter than expected
- The waiting time was as long as expected
- The waiting time was longer than expected

1. **During this stay in the Emergency Department, could you stay physically close to the child/adolescent?**

*Only answer this question if the following conditions are met:*

*Answer was 'The child/adolescent attended the Emergency Department' at question '2' (How did the child's/adolescent’s access to this hospital occur?)*

- The child/adolescent was alone and there were neither relatives nor trusted people with him/her
- Always
- Often
- Sometimes
- Rarely
- Never

1. **Were the Emergency Department medical and nursing staff professional in dealing with the child's/adolescent’s health problem?**

*Only answer this question if the following conditions are met:*

*Answer was 'The child/adolescent attended the Emergency Department' at question '2' (How did the child's/adolescent’s access to this hospital occur?)*

- Always
- Often
- Sometimes
- Rarely
- Never

1. **Did the Emergency Department medical and nursing staff work together to address the health problem of the child/adolescent?**

*Only answer this question if the following conditions are met:*

*Answer was 'The child/adolescent attended the Emergency Department' at question '2' (How did the child's/adolescent’s access to this hospital occur?)*

- Always
- Often
- Sometimes
- Rarely
- Never

1. **How likely are you to recommend this Emergency Department to relatives/friends/acquaintances having children/adolescents with the same health problem as your child/adolescent?**

*Only answer this question if the following conditions are met:*

*Answer was 'The child/adolescent attended the Emergency Department' at question '2' (How did the child's/adolescent’s access to this hospital occur?)*

- 0 = Not at all likely
- 1
- 2
- 3
- 4
- 5
- 6
- 7
- 8
- 9
- 10 = Extremely likely

1. **Do you have any positive or negative comments on your stay in the Emergency Department? If so, use this space.**
2. **At the time of admission to the ward, were you and the child/adolescent welcomed with kindness and courtesy by the ward staff?**

- Very much
- Much
- Enough
- A little bit
- Not at all
- I do not know

1. **Do you have any positive or negative comments on the admission of the child/adolescent to the ward? If so, use this space.**

CHILD’S/ADOLESCENT’S HOSPITALIZATION EXPERIENCE

*It is particularly important that the answers to the following questions, related to the patient's hospitalization experience, are given from the child's/adolescent's point of view.*
*If a person other than the child/adolescent is filling in the questionnaire, please note that these questions should be answered from the perspective of the paediatric patient.*

1. **During this hospital stay, did your physical and psychological condition allow you to talk with nurses and doctors about your health care?**

*Only answer this question if the following conditions are met:*

*The child/adolescent is older than 3 and younger than 18 years.*

- Yes, always
- Yes, sometimes
- No

1. **During this hospital stay, how often were you listened to carefully by…**

*Only answer this question if the following conditions are met:*

*The child/adolescent is older than 6 and younger than 18 years.*

*Answer was NOT 'No' at question '13 ' (During this hospital stay, did your physical and psychological condition allow you to talk with nurses and doctors about your health care?)*

|  | **Never** | **Sometimes** | **Usually** | **Always** |
| --- | --- | --- | --- | --- |
| **doctors?** |  |  |  |  |
| **nurses?** |  |  |  |  |

1. **During this hospital stay, how often were you given explanations (for example about your condition or the procedures performed) in a way that was easy for you to understand by…**

*Only answer this question if the following conditions are met:*

*The child/adolescent is older than 6 and younger than 18 years.*

*Answer was NOT 'No' at question '13 ' (During this hospital stay, did your physical and psychological condition allow you to talk with nurses and doctors about your health care?)*

|  | **Never** | **Sometimes** | **Usually** | **Always** |
| --- | --- | --- | --- | --- |
| **doctors?** |  |  |  |  |
| **nurses?** |  |  |  |  |

1. **During this hospital stay, how often were you encouraged to ask questions by…**

*Only answer this question if the following conditions are met:*

*The child/adolescent is older than 6 and younger than 18 years.*

*Answer was NOT 'No' at question '13 ' (During this hospital stay, did your physical and psychological condition allow you to talk with nurses and doctors about your health care?)*

|  | **Never** | **Sometimes** | **Usually** | **Always** |
| --- | --- | --- | --- | --- |
| **doctors?** |  |  |  |  |
| **nurses?** |  |  |  |  |

1. **During this hospital stay, how often did members of staff talk with and act toward you in a way that was right for your age?**

*Only answer this question if the following conditions are met:*

*The child/adolescent is older than 3 and younger than 18 years.*

*Answer was NOT 'No' at question '13 ' (During this hospital stay, did your physical and psychological condition allow you to talk with nurses and doctors about your health care?)*

- Never
- Sometimes
- Usually
- Always

1. **Do you think that, during this hospital stay, you were treated with respect and dignity by…**

*Only answer this question if the following conditions are met:*

*The child/adolescent is older than 3 and younger than 18 years.*

|  | **Always** | **Often** | **Sometimes** | **Rarely** | **Never** | **I do not know** |
| --- | --- | --- | --- | --- | --- | --- |
| **doctors?** |  |  |  |  |  |  |
| **nurses?** |  |  |  |  |  |  |
| **other ward staff?** |  |  |  |  |  |  |

1. **During this hospital stay, do you think that the ward staff supported you in facing your fears and anxieties?**

*Only answer this question if the following conditions are met:*

*The child/adolescent is older than 3 and younger than 18 years.*

- I did not have fears and anxieties
- Always
- Often
- Sometimes
- Rarely
- Never

1. **During this hospital stay, do you think that the ward staff did everything possible to help you manage your pain?**

*Only answer this question if the following conditions are met:*

*The child/adolescent is older than 3 and younger than 18 years.*

- I did not have any pain
- Always
- Often
- Sometimes
- Rarely
- Never

1. **During this hospital stay, did members of staff involve you as much as you would like in choices related to your care?**

*Only answer this question if the following conditions are met:*

*The child/adolescent is older than 13 and younger than 18 years.*

*Answer was NOT 'No' at question '13 ' (During this hospital stay, did your physical and psychological condition allow you to talk with nurses and doctors about your health care?)*

- Always
- Often
- Sometimes
- Rarely
- Never

1. **Do you have any positive or negative comments on the care you received by the ward staff? If so, use this space.**

*Only answer this question if the following conditions are met:*

*The child/adolescent is older than 3 and younger than 18 years.*

ADULT’S EXPERIENCE

*Please note that this section of the questionnaire is addressed exclusively to the person responsible for the paediatric patient during this hospitalization (e.g. one or both parents, a custodial parent or a legal tutor).*

1. **During this hospital stay, did the ward staff support you in facing your fears and anxieties about the child's/adolescent’s health conditions?**

*Only answer this question if the following conditions are met:*

*Answer was NOT 'Adolescent (14-17 years) alone' at question '1' (Who is filling in the questionnaire?)*

- I did not have fears and anxieties
- Always
- Often
- Sometimes
- Rarely
- Never

1. **During this hospital stay, how often were you given as much privacy as you wanted when discussing the child's/adolescent’s care with members of staff?**

*Only answer this question if the following conditions are met:*

*Answer was NOT 'Adolescent (14-17 years) alone' at question '1' (Who is filling in the questionnaire?)*

- Never
- Sometimes
- Usually
- Always

1. **During this hospital stay, did health professionals involve you as much as you would like in the choices related to the child’s/adolescent’s care?**

*Only answer this question if the following conditions are met:*

*Answer was NOT 'Adolescent (14-17 years) alone' at question '1' (Who is filling in the questionnaire?)*

- Always
- Often
- Sometimes
- Rarely
- Never

1. **During this hospital stay, were the answers given by…**

*Only answer this question if the following conditions are met:*

*Answer was NOT 'Adolescent (14-17 years) alone' at question '1' (Who is filling in the questionnaire?)*

|  | **I did not ask any questions** | **Always** | **Often** | **Sometimes** | **Rarely** | **Never** |
| --- | --- | --- | --- | --- | --- | --- |
| **the child's/adolescent’s doctors to your questions clear?** |  |  |  |  |  |  |
| **the child's/adolescent’s nurses to your questions clear?** |  |  |  |  |  |  |

1. **During this hospital stay, how often did members of staff keep you informed about the condition of the child/adolescent and what was being done for him/her?**

*Only answer this question if the following conditions are met:*

*Answer was NOT 'Adolescent (14-17 years) alone' at question '1' (Who is filling in the questionnaire?)*

- Never
- Sometimes
- Usually
- Always

1. **During this hospital stay, did you receive *clear* information on risks and benefits, before giving your consent to the child's/adolescent’s treatment?**

*Only answer this question if the following conditions are met:*

*Answer was NOT 'Adolescent (14-17 years) alone' at question '1' (Who is filling in the questionnaire?)*

- Very much
- Much
- Enough
- A little bit
- Not at all
- I do not remember I received any information on risks and benefits

1. **How would you rate the ability of the ward medical and nursing staff to work together?**

*Only answer this question if the following conditions are met:*

*Answer was NOT 'Adolescent (14-17 years) alone' at question '1' (Who is filling in the questionnaire?)*

- Very good
- Good
- Sufficient
- Poor
- Very poor
- I do not know

1. **During this hospital stay, how often did members of staff check the child's/adolescent’s identity before administering any drugs or starting any diagnostic tests or therapies?**

*Only answer this question if the following conditions are met:*

*Answer was NOT 'Adolescent (14-17 years) alone' at question '1' (Who is filling in the questionnaire?)*

- Always
- Often
- Sometimes
- Rarely
- Never

1. **Do you have any positive or negative comments on the care the child/adolescent received by the ward staff? If so, use this space.**

HOSPITAL ENVIRONMENT

1. **Was the ward where the child/adolescent stayed (room, bathroom, corridors, other common spaces, etc.) noisy?**

- Not at all
- A little bit
- Enough
- Much
- Very much

1. **Was the ward where the child/adolescent stayed (room, bathroom, corridors, other common spaces, etc.) clean?**

- Very much
- Much
- Enough
- A little bit
- Not at all

1. **During this hospital stay, was the temperature in the ward (room, bathroom, corridors, other common spaces, etc.) adequate?**

- Always
- Often
- Sometimes
- Rarely
- Never

1. **During this hospital stay, was the food catering service offered to the child/adolescent satisfying?**

- Always
- Often
- Sometimes
- Rarely
- Never
- I do not know

1. **Hospitals can have things like toys, books, mobiles, and games for children from newborns to teenagers. During this hospital stay, did the hospital have things available for the child/adolescent that were right for the child’s/adolescent’s age?**

- Yes, definitely
- Yes, somewhat
- No

1. **Do you have any positive or negative comments on the comfort of the ward where the child/adolescent stayed? If so, use this space.**

LEAVING THE HOSPITAL

1. **Before the child/adolescent left the hospital, did a member of staff ask you if you had any concerns about whether the child/adolescent was ready to leave?**

- Yes, definitely
- Yes, somewhat
- No

1. **Before the child/adolescent left the hospital, did a member of staff talk with you as much as you wanted about how to care for the child/adolescent’s health after leaving the hospital?**

- Yes, definitely
- Yes, somewhat
- No

1. **A child/adolescent’s regular activities can include things like eating, bathing, going to school, or playing sports. Before the child/adolescent left the hospital, did a member of staff explain in a way that was easy to understand when the child/adolescent could return to his or her regular activities?**

- Yes, definitely
- Yes, somewhat
- No

1. **Before the child/adolescent left the hospital, did a member of staff tell you that the child/adolescent should take any new medicines that he or she had not been taking when this hospital stay began?**

- Yes
- No, because the child/adolescent did not need it

1. **Before the child/adolescent left the hospital, did a member of staff explain in a way that was easy to understand…**

*Only answer this question if the following conditions are met:*

*Answer was 'Yes' at question '41' (Before the child/adolescent left the hospital, did a member of staff tell you that the child/adolescent should take any new medicines that he or she had not been taking when this hospital stay began?)*

|  | **Yes, definitely** | **Yes, somewhat** | **No** |
| --- | --- | --- | --- |
| **how the child/adolescent should take these new medicines after leaving the hospital?** |  |  |  |
| **about possible side effects of these new medicines?** |  |  |  |

1. **Before the child/adolescent left the hospital, did a member of staff explain in a way that was easy to understand what symptoms or health problems to look out for after the child/adolescent left the hospital?**

- There were no symptoms or health problems to monitor
- Yes, definitely
- Yes, somewhat
- No

1. **Before the child/adolescent left the hospital, did you get information in writing?**

- No, I did not receive any written information
- Yes, I received information in writing on the drugs to administer to the child/adolescent
- Yes, I received information in writing on the symptoms or health problems to monitor once back home
- Yes, I received information in writing on the management of the child's/adolescent’s daily activities
- Yes, I received the discharge letter
- Other:

1. **Was the information written in the discharge letter clear?**

*Only answer this question if the following conditions are met:*

*Answer was 'Yes, I received the discharge letter' at question '44' (Before the child/adolescent left the hospital, did you get information in writing?)*

- Very much
- Much
- Enough
- A little bit
- Not at all
- I do not know

1. **Was the information written in the received materials (except the discharge letter) clear?**

*Only answer this question if the following conditions are met:*

*Answer was NOT 'Yes, I received the discharge letter' at question '44' (Before the child/adolescent left the hospital, did you get information in writing?)*

- Very much
- Much
- Enough
- A little bit
- Not at all
- I do not know

1. **Before the child/adolescent left the hospital, did you receive any specific training to learn how to use specific medical devices or drugs, necessary for managing the child/adolescent once back home?**

- Yes
- No, there was no need
- No, but we needed it

1. **How useful was this specific training in order to personally take care of the child/adolescent once back home?**

*Only answer this question if the following conditions are met:*

*Answer was 'Yes' at question '47' (Before the child/adolescent left the hospital, did you receive any specific training to learn how to use specific medical devices or drugs, necessary for managing the child/adolescent once back home?)*

- Very much
- Much
- Enough
- A little bit
- Not at all

OVERALL EVALUATION

1. **Overall, how would you rate the care that the child/adolescent received in the ward?**

- Very good
- Good
- Sufficient
- Poor
- Very poor
- I do not know

1. **How likely is it you would recommend this ward to relatives/friends/acquaintances having children/adolescents with the same health problem as that of your child/adolescent?**

- 0 = Not at all likely
- 1
- 2
- 3
- 4
- 5
- 6
- 7
- 8
- 9
- 10 = Extremely likely

1. **Help us give value to the people who took care of the child/adolescent: would you like to indicate any people who impressed you for the way they treated you and the child/adolescent? If so, use this space.**

ONCE BACK HOME

1. **In general, how do you consider the child's/adolescent’s current health status?**

- Excellent
- Very good
- Good
- Fair
- Poor

1. **Once back home, did the child/adolescent need home care from a nurse or other health care provider?**

- Yes, he/she did, and the health system provided him/her with it
- Yes, he/she did, but I had to find the health professionals and pay for them
- No, he/she did not need it
- Other

ADULT’S CHARACTERISTICS

1. **Your gender:**

*Only answer this question if the following conditions are met:*

*Answer was 'Custodial parent' or 'Custodial parent together with the adolescent (14-17 years)' or 'Legal guardian' or 'Legal guardian together with the adolescent (14-17 years)' at question '1' (Who is filling in the questionnaire?)*

- Female
- Male

1. **Age of mother / custodial mother / legal guardian:**

- <18 years
- 18-24 years
- 25-31 years
- 32-38 years
- 39-45 years
- 46-50 years
- 51-55 years
- 56-60 years
- 61-65 years
- > 65 years
- There is not the mother

1. **Citizenship of mother / custodial mother / legal guardian:**
2. **Is the mother / custodial mother / legal guardian employed?**

- Yes
- No

1. **Education of mother / custodial mother / legal guardian:**

** This question is characterized by a specific response scale reflecting the Italian schooling system in the questionnaire in use at Meyer*

- No formal education
- Basic education (9 years)
- Secondary education (12 years)
- Academic or professional higher education
- Master and/or doctoral studies

1. **Age of father / custodial father / legal guardian:**

- <18 years
- 18-24 years
- 25-31 years
- 32-38 years
- 39-45 years
- 46-50 years
- 51-55 years
- 56-60 years
- 61-65 years
- > 65 years
- There is not the father

1. **Citizenship of father / custodial father / legal guardian:**
2. **Is the father / custodial father / legal guardian employed?**

- Yes
- No

1. **Education of father / custodial father / legal guardian:**

** This question is characterized by a specific response scale reflecting the Italian schooling system in the questionnaire in use at Meyer*

- No formal education
- Basic education (9 years)
- Secondary education (12 years)
- Academic or professional higher education
- Master and/or doctoral studies

1. **During the child's/adolescent’s hospital stay, how much of the time were you at the hospital?**

*Only answer this question if the following conditions are met:*

*Answer was NOT 'Adolescent (14-17 years) alone' at question '1' (Who is filling in the questionnaire?)*

- None of the hospitalization time
- Less than a half of the hospitalization time
- Half of the hospitalization time
- More than a half of the hospitalization time
- All or nearly all of the hospitalization time
